# Transcription Regulation by Class III Histone Deacetylases (HDACs)—Sirtuins

**DOI:** 10.4137/tog.s483

**Published:** 2008-04-23

**Authors:** Yan Dai, Douglas V. Faller

**Affiliations:** Cancer Research Center and Department of Medicine, Boston University School of Medicine, Boston, Massachusetts 02118, U.S.A

**Keywords:** Sirtuins, SIRT1, transcription, acetylation, deacetylation, HDAC

## Abstract

Sirtuins are NAD^+^-dependent histone deacetylases (Class III HDACs). Recently, Sirtuins have been shown to play important roles, both direct and indirect, in transcriptional regulation. This transcriptional control, through incorporation of Sirtuins into transcription complexes and deacetylation of histones locally at gene promoters, or direct interaction with specific transcription factors, is central to the participation of Sirtuins in multiple diverse processes, including aging, apoptosis, hormone responses, stress tolerance, differentiation, metabolism and development. Here we review the contribution of the Sirtuin family, at multiple molecular levels, to transcriptional regulation.

## Introduction

The modification of histones and transcription factors by acetylation and deacetylation play a central role in the control of transcription. Acetylation of histones is postulated to increase DNA accessibility through the neutralization of the positive charges of lysine residues. This modification correlates generally with transcriptional activation. Histone acetylation is catalyzed by histone acetyl transferases (HATs), whereas the reverse reaction is performed by histone deacetylases (HDACs). HATs and HDACs participate in the genome-wide turnover of acetyl groups on histones and, in addition, on many transcription factors. Through their physical interactions with DNA sequence-specific transcription factors, they are also targeted to specific promoters, where they locally modify histones or transcription factors or transcriptional co-activators/co-repressors, and thus can regulate gene transcription at multiple molecular levels.

HATs and HDACs are classified into numerous families that are often conserved from yeast to humans. Histone deacetylases (HDACs) are divided into three classes. Class I members (HDACs 1, 2, 3, 8, 11) are transcriptional co-repressors homologous to yeast RPD3 [1]. HDAC class II members (HDACs 4, 5, 6, 7, 9, 10) have domains similar to yeast HDA1 [2]. Class III histone deacetylases (Sirtuins) are structurally distinct from class I and II HDACs, and are homologues of the yeast silent information regulator 2 (Sir2) [3, 4]. Class III HDACs are NAD^+^-dependent histone deacetylases, wherein their deacetylase activity is controlled by the cellular [NAD^+^]/[NADH] ratio. The mammalian homologues of the Sirtuin family proteins consist of seven members, SIRT1—SIRT7 [5]. SIRT1 (also known as Sir2α) is the closest homologue of yeast Sir2 and has been the most extensively studied.

All seven Sirtuins are ubiquitously expressed in humans, and histones H1, H3 and H4 appear to be the histone protein targets of the class III histone deacetylases. A growing number of non-histone substrates have also been shown to be deacetylated by Sirtuin family members, greatly expanding the potential biological roles of Sirtuins. These non-histone Sirtuin substrates range in class from structural to adhesion to signaling molecules, and the activity of these protein targets of Sirtuins, or their sub-cellular localization, or their association with other proteins, can be modified by their acetylation state. Non-histone Sirtuin targets related to transcriptional control include many DNA-binding transcription factors, such as the forkhead box type O transcription factors (FOXO) [6–10], p53 [11–13], nuclear factor-κB (NFκB) [14] and the peroxisome proliferator activated receptor-γ (PPARγ) [15], the androgen receptor [16, 17] and their co-regulatory molecules. In this review, we will review the basic underlying principles of Sirtuin family member actions on transcriptional regulation and the major advances that have occurred in this field in recent years.

## Regulation of Transcription Factor Function by Sirtuins

**Figure 1:**
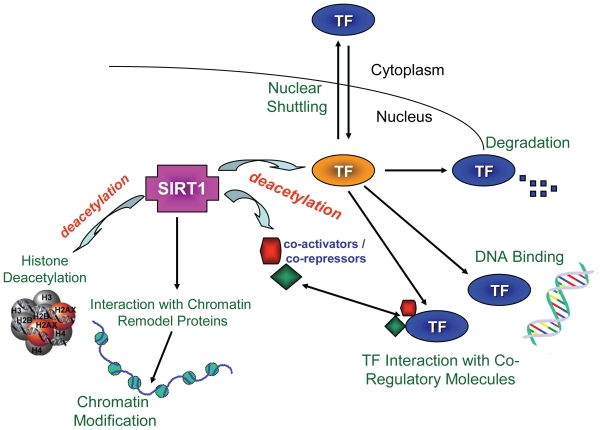
Sirtuins regulates multiple aspects of transcription. SIRT1 can deacetylate transcription factors (TF) and regulate their functions by altering their sub-cellular location, changing their expression level, altering their binding to DNA and changing their interactions with regulatory proteins. SIRT1 can also directly deacetylate co-repressor or co-activator, histone and interact with chromatin remodeling proteins to regulate transcription.

Numerous mammalian transcription factors and their co-regulatory proteins have been identified as targets of the Sirtuins gene products (Table 1). The deacetylation of transcription factors by Sirtuins can regulate their activities in a number of ways, including: (i) altering their sub-cellular location; (ii) changing their expression level; (iii) altering their binding to DNA; and, (iv) changing their interactions with regulatory proteins (Fig. 1). (Regulation of transcription factors by Sirtuins often involves more than one of these mechanisms simultaneously).

### Alteration of the sub-cellular localization of transcription factors by Sirtuins

Nucleocytoplasmic shuttling of transcription factors is an important mechanism in transcription regulation. The acetylation or deacetylation of a transcription factor can directly regulate the accessibility of nuclear localization signals (NLSs) and nuclear export signals (NESs) to nuclear import and export proteins. SIRT1 is implicated in regulating the sub-cellular localization of several transcription factors, including members of the FOXO transcription factor family. However, the precise molecular mechanism regulating the shuttling of FOXO by Sirtuins is not yet clear.

Forkhead box O (FOXO) transcription factors, including FOXO1, FOXO3a, FOXO4 and FOXO6, the mammalian orthologs of *Caenorhabditis elegans* DAF-16, are emerging as a central family of proteins that modulate the expression of genes involved in apoptosis, cell cycle progression, DNA damage repair, responses to oxidative stress, cell differentiation, glucose metabolism and other cellular functions [18, 19]. FOXO proteins are regulated by post-translational modifications, including phosphorylation and acetylation [20, 21]. In growing cells, FOXO proteins are predominantly cytoplasmic, and nuclear import of FOXO proteins is triggered by oxidative stress or DNA damage. SIRT1 deacetylates FOXO1 at Lys-242, -245, and -262, overrides the phosphorylation-dependent nuclear exclusion of FOXO1 caused by growth factors, and induces the nuclear translocation of FOXO1 [7]. FOXO1 is readily diffusible within the nucleus under normal conditions but becomes restricted within a nuclear subdomain following treatment with the prototypical Sirtuin agonist resveratrol, or after exposure to oxidative stress, leading to the expression of FOXO1 target genes, and ultimately inducing gluconeogenesis and glucose release from hepatocytes [7]. (FOXO protein transcriptional activity and stability are also directly altered through deacetylation of FOXO by SIRT1, and these additional levels of FOXO regulation by SIRT1 will be discussed below).

SIRT2, another member of the class III HDAC family, regulates FOXO1 translocation in 3T3-L1 adipocytes [22]. SIRT2, a cytoplasmic Sirtuin, is the most abundant Sirtuin in adipocytes. SIRT2 directly associates with, and deacetylates, FOXO1, leading to changes in FOXO1 phosphorylation, enhanced nuclear localization, and inhibition of adipogenesis. Thus, SIRT2 acts as an important regulator of adipocyte differentiation through modulation of FOXO1 acetylation/phosphorylation and activity [22].

FOXO3 localization is regulated by SIRT1; translocation of FOXO3 from the cytoplasm to the nucleus is induced by SIRT1 in response to oxidative stress [8]. In the presence of growth factors and the absence of stress, FOXO3 is cytoplasmic. In cells subjected to various stresses, including oxidative stress, FOXO3 relocates to the nucleus. SIRT1 forms a complex with deacetylated FOXO3 in the nucleus, inducing cell-cycle arrest and resistance to oxidative stress, but simultaneously inhibiting the ability of FOXO3 to induce apoptosis [8].

The examples described above demonstrate that deacetylation of FOXO transcription factors by SIRT1 promotes their nuclear translocation. However, FOXO regulation by SIRT1 appears to occur at multiple levels. SIRT1 has been shown to associate with, and deacetylate, FOXO proteins in the nucleus. A FOXO3 mutant with three Akt phosphorylation sites mutated to alanine has been shown to reside permanently in the nucleus, but does not interact with SIRT1 in the absence of stress signals [8], suggesting that nuclear localization of FOXO3 alone is not sufficient to promote an interaction with nuclear SIRT1, and that some form of phosphorylation or other modification of FOXO3 might be required for the interaction between FOXO3 and SIRT1. Although SIRT1 is reported to be predominantly nuclear in localization, SIRT1 has also been found recently to also be resident in the cytoplasm, and can shuttle between the nucleus and cytoplasm [23–28]. Whether or not cytoplasmic SIRT1 plays a role in FOXO3 localization requires further study.

### The control of transcription factor stability by Sirtuin-mediated deacetylation

The control of the transcription factor protein levels is another important mechanism in transcription regulation. The acetylation status of the lysine residues on a protein can exert important direct regulatory control of that protein’s stability [29]. Acetylation of certain transcription factors on lysine residues protects them from degradation, thereby promoting their transcriptional activity [30, 31]. Conversely, the deacetylation of these transcription factors by SIRT1 adversely affects their stability by regulating the covalent attachment of ubiquitin to lysine residues. Lysine acetylation is one mechanism controlling the stability of the Smad7 transcription factor; SIRT1-dependent deacetylation of Smad7 promotes its ubiquitination and degradation [32]. Other examples in which SIRT1 has been shown to regulate transcription factor protein stability include FOXO [33], androgen receptor [16], PGC-1α [34] and p53 [35].

The Smad family of signal transduction molecules are components of a critical intracellular pathway that transmits TGF-β signals from the cell surface into the nucleus. Once in the nucleus, Smads target a variety of DNA-binding proteins to regulate transcriptional responses. SIRT1 directly interacts with the N-terminus of Smad7 and thereby regulates transforming growth factor β (TGFβ)-induced apoptosis in renal glomerular mesangial cells [32]. SIRT1 reverses histone acetyl-transferase (HAT) p300-mediated acetylation of two lysine residues (Lys-64 and -70) on Smad7. In mesangial cells, the expression level of Smad7 is reciprocally reduced by SIRT1 over-expression and increased by SIRT1 knockdown. SIRT1-mediated deacetylation of Smad7 enhances Smad ubiquitination regulatory factor 1 (Smurf1)-mediated ubiquitin proteasome degradation, which contributes to the low levels of Smad7 expression in SIRT1-overexpressing mesangial cells. SIRT1 over-expression attenuates Smad7-induced mesangial cell apoptosis, whereas SIRT1 knockdown enhances this apoptotic response [32].

While FOXO transcription factors are regulated by Sirtuins predominantly *via* reversible changes in their sub-cellular localization, the stability of FOXO proteins represents an additional and irreversible level of regulation for this family of transcription factors. Degradation of FOXO transcription factors is mediated by the ubiquitin-proteasome pathway. In addition to phosphorylation modifications, acetylation also plays a role in the control of ubiquitin-mediated FOXO protein degradation. Acetylation of FOXO1 prevents its ubiquitin-dependent degradation [33]. Our unpublished data demonstrates that SIRT1 down-regulation in prostate cancer cell lines increases the levels of FOXO3a protein, suggesting a role for deacetylation by SIRT1 in ubiquitin-mediated FOXO3a protein degradation.

The androgen receptor (AR) is a ligand-regulated modular nuclear receptor governing prostate cancer cellular proliferation, differentiation, and apoptosis in response to androgens. SIRT1 binds to, and deacetylates, the androgen receptor at a conserved lysine motif, down-regulating its levels in the cell, and repressing androgen-induced AR transcriptional activity [16].

The protein level of the p53 tumor suppressor is regulated by SIRT1. p53 is stabilized by acetylation of Lys-382, and this residue is a known target of SIRT1-mediated deacetylation [35]. As p53 plays a central role in critical cellular processes and fates, including DNA repair, apoptosis, cell cycle arrest, senescence and transformation, multiple pathways regulating cell fate may operate through Sirtuins on p53. For example, Nampt, a longevity protein, extends the lifespan of human smooth muscle cells by activating SIRT1 and restraining the accumulation of p53 [35]. The transcriptional activity of p53 is also regulated by Sirtuins at the level of DNA-binding, and the transcription of the *SIRT1* gene is reciprocally regulated by p53, as discussed below.

One mechanism of transcriptional regulation involves the replacement of core histones with more specialized variants, and Sirtuins has a role in regulating the stability of variant histones [24]. H2A.z is one such histone variant and serves as a transcriptional regulator essential for development of mammals [36]. Histone variant H2A.z is up-regulated during cardiac hypertrophy and is required for this pathological response, and H2A.z stability and the extent of myocyte cell growth and apoptosis are moderated by Sir2α (the mouse homologue of yeast Sir2) [24]. Over-expression of Sir2α specifically induces down-regulation of H2A.z *via* a NAD^+^-dependent activity. This effect is reversed by the proteasome inhibitor epoxomicin, suggesting a Sir2α-mediated ubiquitin/proteasome-dependent mechanism for degradation of H2A.z. Sir2α directly deacetylates H2A.z, and Lys-115 and a conserved, ubiquitinatable Lys-121 are critical for Sir2α-mediated degradation. Sir2α is therefore involved in the deacetylation of H2A.z, leading to the ubiquitination of Lys-115 and Lys-121 and the consequent degradation of H2A.z *via* a ubiquitin/proteasome-dependent pathway [24].

### Regulation of transcription factor binding to DNA by Sirtuins

The binding of transcription factors to gene promoters can be regulated by Sirtuins-mediated acetylation. As one example, the acetylation of lysine residues in the C-terminal regulatory domain of p53 enhances the sequence-specific binding of p53. SIRT1 deacetylates p53 at Lys-382 in the C-terminal domain and represses its transcriptional activity [11, 37]. As a consequence, SIRT1 represses p53-dependent apoptosis in response to DNA damage and oxidative stress, and promotes cell survival under cellular stress induced by agents such as etoposide or irradiation. Interestingly, as SIRT1 transcription is directly repressed by p53, SIRT1-induced repression of p53 function is likely to result in feed-forward induction of SIRT1 protein levels. p73 is also a target of SIRT1, and direct deacetylation of p73 by SIRT1 changes the DNA-binding ability of this factor and reduces its transcriptional activity [38].

Binding of the central transcription factor and tumor suppressor E2F1 to its cognate gene promoter elements and their subsequent transcriptional activation, are stimulated by acetylation through PCAF. SIRT1 deacetylates E2F and suppresses its transcriptional activity, most likely through suppression of its DNA-binding activity [39]. As the *SIRT1* gene is a transcriptional target of E2F1, this may provide some auto-regulation of SIRT1 levels in the cell, with SIRT1 repressing its own transcription through E2F (while stimulating its own transcription through inhibition of p53 activity).

SIRT1 physically interacts with the RelA/p65 subunit of NFκB and inhibits NFκB-induced transcription, by deacetylating RelA/p65 at Lys-310. Increases in SIRT1 activity delays TNFα-induced recruitment of NFκB complexes containing RelA/p65 and p50 proteins to NFκB-regulated gene promoters [14].

SIRT1 is a binding partner for the retinoblastoma tumor suppressor protein (Rb) and its family members, p107 and p130, and SIRT1 is an *in vitro* and *in vivo* deacetylase for the Rb protein [40]. Active Rb is acetylated in multiple residues and inactivation of Rb involves deacetylation of lysine residues within Rb. p300 catalyzes the acetylation and activation of Rb, whereas SIRT1 is a potent deacetylase for Rb. The deacetylated lysine residues within Rb form a domain similar to the SIRT1-targeted domain of the p53 tumor suppressor protein. The deacetylation of Rb by SIRT1 results in the inhibition of Rb tumor suppressor activity.

Sirtuins have been shown to directly deacetylate members of the FOXO transcription factor family and alter their transcriptional activity. Since many FOXO acetylation sites are located within the DNA-binding domain of the molecule, it is possible that acetylation of FOXO interferes with FOXO binding to DNA and thereby inhibits FOXO-dependent transcription. For example, SIRT1 has been shown to preferentially deacetylate FOXO1 at K242, K245, and K262, thereby altering its DNA-binding ability, and activating the transcriptional function of FOXO1 in a deacetylase-activity dependent manner [41, 42]. As another example, SIRT2 binds to FOXO3 and reduces its acetylation levels. SIRT2 thereby increases FOXO3 DNA-binding activity and elevates the expression of FOXO target genes. As a consequence, SIRT2 activation, through FOXO3, decreases cellular levels of reactive oxygen species and induces cell cycle arrest. Furthermore, as Bim is a pro-apoptotic factor, SIRT2 promotes cell death when cells are subjected to severe stress [43].

Sirtuins can also regulate transcription by affecting the activity of the basal transcription machinery. Acetylation of TAFI68, a subunit of the RNA polymerase I complex, enhances the binding of TAFI68 to *rRNA* gene promoters and induces *rRNA* gene transcription in mammals. Sir2a deacetylates TAFI68 and decreases the binding of TAFI68 to rDNA promoters and represses rDNA gene transcription [44]. Thus, TAFI68 represents a new class of Sirtuin substrates whose site-specific DNA-binding activity is decreased by the action of these class III HDACs.

### Regulation of the interactions of transcription factors with their co-regulators by Sirtuins

In addition to regulating transcription factors at the levels of the sub-cellular localization, protein stability and DNA-binding ability, SIRT1 can also modulate intrinsic transcription factor activity through alteration of the affinity of the factors for their co-activators, co-repressors or the general transcription machinery. Sirtuins could modulate such interactions directly, by deacetylating residues on the transcription factors or the co-regulatory molecules which form parts of interacting surfaces, thereby blocking or facilitating interactions, or indirectly, with deacetylation inducing a conformational change in another part of the molecule which unmasks or masks binding surfaces.

SIRT1-mediated deacetylation enhances FOXO1 and CCAAT/enhancer-binding protein alpha (C/EBPα) interactions, thereby promoting the transcription of adiponectin, an adipose tissue-derived hormone which plays an important role in maintaining energy homeostasis [45]. C/EBPα acts as a co-activator of FOXO1, which accesses the adiponectin promoter through two FOXO1-binding sites. FOXO1 interacts with C/EBPα to form a transcription complex and up-regulates adiponectin gene transcription. In addition to enhancing FOXO1—C/EBPα interactions [45], SIRT1 also acts to increase adiponectin transcription in adipocytes by activating FOXO1 directly through its deacetylation (see above).

Transcription factor PPARγ is a key regulator of adipogenesis and fat storage, controlling the expression of many adipocyte-specific genes. SIRT1 actively represses PPARγ activity *via* enhancing PPARγ docking with two of its co-repressors, NcoR (nuclear receptor co-repressor) and SMRT (silencing mediator of retinoid and thyroid hormone receptor). Hence, SIRT1 acts as a co-repressor of PPARγ-mediated transcription, attenuating adipogenesis [15].

The androgen receptor (AR) is a ligand-regulated modular nuclear receptor governing prostate cancer cellular proliferation, differentiation, and apoptosis in response to androgens. We have demonstrated ligand-dependent recruitment of SIRT1 as a co-repressor into a transcription complex [17], the first example of conditional recruitment of SIRT1 into a transcription complex. SIRT1 recruitment is required for androgen antagonist-mediated transcriptional repression and growth suppression of AR-responsive genes. Androgen antagonist-bound androgen receptor (AR) recruits SIRT1 and nuclear receptor co-repressor (NCoR) to AR-responsive promoters and deacetylates histone H3 locally at these promoters.

### Regulation of transcription factor co-regulator protein function by Sirtuins

In addition to direct regulation of transcription factors themselves, increasing evidence suggests that co-regulatory factors, including p300, PGC1 and CTIP1/CTIP2, are also directly targeted by Sirtuins.

The p300 protein serves as a rate-limiting transcriptional co-integrator of diverse transcription factors, either activating or repressing transcription through its modular subdomains. SIRT1 physically interacts with, and represses transactivation by, p300. Two residues within the CRD1 domain of p300 (Lys-1020 and Lys-1024) are required for repression by SIRT1 and serve as substrates for SIRT1 deacetylation. Because p300 is a gating transcriptional cofactor, deacetylation and repression of p300 by SIRT1 may serve as an important integration point during metabolic processes and cellular differentiation [46].

The transcriptional co-activator PGC-1α plays an important role in hepatic and systemic glucose and lipid metabolism and respiration, as well as caloric restriction. In mammals, maintenance of energy and nutrient homeostasis during food deprivation is accomplished through an increase in mitochondrial fatty acid oxidation in peripheral tissues. PGC-1α is an important component driving this cellular oxidative process, and SIRT1 interacts with PGC-1α and deacetylates it at multiple lysine residues, to activate PGC-1α-mediated transcription. Fasting induces PGC-1α deacetylation in skeletal muscle and SIRT1 deacetylation of PGC-1α is required for activation of mitochondrial fatty acid oxidation genes. SIRT1 therefore serves as a functional regulator of PGC-1α regulating a metabolic gene transcription program of mitochondrial fatty acid oxidation. SIRT1, *via* deacetylation of PGC-1α, activates gluconeogenesis and represses glycolysis in the liver [47, 48]; protects mice against diet-induced obesity and insulin resistance [49]; promotes the activation of mitochondrial fatty acid oxidation genes [50] and regulates cellular oxygen consumption [51]. Many of the metabolic changes induced by SIRT1 knockdown or SIRT1 over-expression are dependent upon PGC-1α [48]. Treatment with resveratrol (a SIRT1 activator) protects mice against diet-induced obesity and insulin resistance [49] (although SIRT1 is not the only target of resveratrol, and has not been shown to be the critical functional target of the drug in these studies). This regulation of energy and metabolic homeostasis by SIRT1 has implications for understanding the consequences of selective nutrient adaptation and how it might impact lifespan or metabolic diseases such as obesity and diabetes [50].

SIRT3 is localized to the mitochondrial inner membrane. Caloric restriction activates SIRT3 expression in both white and brown adipose tissue. SIRT3 interacts with and deacetylates PGC-1α to activate mitochondria functions, and plays an important role in adaptive thermogenesis in brown adipose tissue [52]. Enforced expression of SIRT3 in HIB1B brown adipocytes enhances the expression of PGC-1α, the uncoupling protein UCP1, and a series of mitochondria-related genes. In addition, SIRT3 stimulates CREB phosphorylation, which reportedly activates the PGC-1α promoter directly. Functionally, sustained expression of SIRT3 decreases membrane potential and reactive oxygen species production, while increasing cellular respiration [52].

Chicken ovalbumin upstream promoter transcription factor II (COUP-TF II)-interacting proteins 1 and 2 (CTIP1 and CTIP2) enhance transcriptional repression mediated by COUP-TF II and have been implicated in hematopoietic cell development and malignancies. CTIP1 and CTIP2 are also sequence-specific DNA-binding proteins that repress transcription through direct, COUP-TF-independent binding to a GC-rich response element. SIRT1 interacts directly with CTIP1/CTIP2 and is recruited to specific promoter elements in a CTIP1/CTIP2-dependent manner. SIRT1 enhances the deacetylation of template-associated histones H3/H4 in CTIP-transfected cells (see below), and stimulates CTIP-dependent transcriptional repression [53, 54].

### Sirtuins as regulatory components of transcription factor complexes

Recent reports now clearly demonstrate that SIRT1 can form complexes with certain transcription factors bound to promoter elements, and regulate their transcriptional activity. The biochemical mechanisms underlying of transcription regulation by SIRT1 in these complexes are not clearly defined in every case, and may include local histone deacetylation at the promoter, direct deacetylation of other members of the complex, or a change the ratio of co-activators/co-repressors within the transcription complex (and more than one such SIRT1-mediated mechanism may be simultaneously operative within the same complex).

The basic helix—loop—helix (bHLH) superfamily of transcription factors comprises a large number of proteins which play important roles as activators or repressors in diverse aspects of development. SIRT1 physically associates with the human bHLH repressor proteins hHES1 and hHEY2, both *in vitro* and *in vivo*, and enhances the transcriptional repression mediated by these bHLH repressors [55].

Tumor suppressor HIC1 (hypermethylated in cancer 1) encodes a zinc-finger transcription factor that is essential for mammalian development, which is epigenetically silenced in many human tumors and is involved in a complex pathway regulating p53 tumor suppression activity. Two deacetylases, SIRT1 and HDAC4, interact and form a dual-deacetylase complex with the transcriptional repressor HIC1, enhancing the transcriptional suppressor effect of HIC1 [56]. The activity of HIC1 as a transcriptional repressor is inhibited by its acetylation by the HATs CBP/p300. SIRT1 deacetylates HIC1 at the same lysine residues, and promotes its sumoylation, resulting in restoration of the repressor activity of HIC1 [56]. In a autoregulatory loop analogous to SIRT1 suppression of E2F transcriptional activity and p53 transcriptional repressor activity (discussed above), because HIC1 is a transcriptional repressor of the *SIRT1* gene, one result of SIRT1 interactions with HIC1-containing transcriptional repressor complexes is down-regulation of *SIRT1* gene expression [57].

SIRT1 acts predominantly as a co-repressor of gene expression through protein deacetylation. However, there is evidence that SIRT1 can also serve as a co-activator, positively-regulating gene expression. As one example, the human immunodeficiency virus (HIV) Tat protein is acetylated by the transcriptional co-activator p300. SIRT1 recycles Tat to its unacetylated form and acts as a transcriptional co-activator during Tat transactivation [58]. Tat and SIRT1 association in a DNA-bound transcription factor complex synergistically activates the HIV promoter.

## Regulation of Transcription at the Nucleosomal Level by Sirtuins

In addition to its pleiotropic effects on specific non-histone target proteins, SIRT1 also regulates transcription by deacetylating histones and interacting with chromatin-remodeling complexes.

### Deacetylation of histones at gene promoters by Sirtuins

A diverse array of post-translational modifications within the tail domains of histones have been shown to regulate chromatin structure and hence regulate gene transcription. SIRT1 preferentially deacetylates H3-K9, H3-K14 and H4-K16 *in vitro* and *in vivo* [59].

Certain transcription factors, including CTIP1, CTIP2, and AR, can recruit SIRT1 to target gene promoters, with resulting local deacetylation of either histones H3 or H4, thereby repressing gene transcription.

As discussed above, CTIP1 (BCL11A) and CTIP2 (BCL11B) are transcriptional repressors which bind directly to a GC-rich motif and are also indirectly recruited to promoter elements *via* interaction with the orphan nuclear receptor, chicken ovalbumin upstream promoter transcription factor II (COUP-TF II). SIRT1 interacts directly with CTIP1 and CTIP2 and is recruited to the promoter template in a CTIP1/CTIP2-dependent manner, leading to transcriptional repression. Chromatin immunoprecipitation assays revealed that expression of CTIP1 or CTIP2 in mammalian cells results in deacetylation of histones H3 and/or H4 associated with the promoter region, in a SIRT1-dependent mechanism. CTIP-mediated transcriptional repression, as well as deacetylation of histone H3/H4 in CTIP1-transfected cells, is partially reversed by nicotinamide, an inhibitor of class III histone deacetylases [53, 54].

In addition to modulating the levels of AR by direct deacetylation of this transcription factor, discussed above, ligand-dependent recruitment of SIRT1 into AR transcriptional repressor complexes on AR-responsive promoters results in the deacetylation of histone H3 locally [17].

SIRT2 also plays a role in histone deacetylation, in particular at histone residue H4-K16. SIRT2 is predominantly localized to the cytoplasmic compartment, where it serves to deacetylate α-tubulin. During mitosis, however, SIRT2 shuttles to the nucleus and deacetylates histone H4-K16 to promote chromatin condensation [28].

SIRT3 can also deacetylate Histone H4-K16 and repress transcription in a gene-specific manner. SIRT3 exists in two forms, a full-length protein and a processed polypeptide lacking 142 amino acids at the N-terminus. Both the full-length and processed forms of SIRT3 deacetylate H4-K16 *in vivo* and repress gene transcription [60].

Tumor suppressor gene (TSG) silencing is a hallmark of cancer. Epigenetic mechanisms of silencing of tumor suppressor genes in some cases involves dense hypermethylation of 5′ CpG islands and local hypoacetylation of Lys-9 and -14 on histone H3 (H3-K9 and H3-K14, respectively). Recently, SIRT1 has been shown to localize to the promoters of several aberrantly-silenced TSGs in which 5′ CpG islands are densely hypermethylated, but not to these same promoters in cell lines in which the promoters are not hypermethylated and the genes are expressed. Inhibition of SIRT1 in breast and colon cancer cells causes increases in H4-K16 and H3-K9 acetylation at endogenous promoters and re-expression of silenced TSGs, despite full retention of promoter DNA hypermethylation. SIRT1, therefore, comprises a new component of epigenetic TSG silencing that may potentially link some of the epigenetic changes associated with aging with those found in cancers, and provides new directions for potential therapeutic targeting these important genes for re-expression [61].

In addition to deacetylation of H3 and H4, the linker histone H1 may also serve as a target of SIRT1 in genome-wide transcriptional repression [62]. Histone H1 is acetylated *in vivo* at Lys-26 and can be deacetylated by SIRT1. SIRT1 physically interacts with histone H1, and this interaction recruits histone H1 to establish repressive chromatin domains. Upon induction of SIRT1 activity, there is a reduction in H4-K16 acetylation in euchromatic chromosomal regions. This effect is accompanied by an enrichment of histone H1 at specific promoters, the spreading of heterochromatin marks like tri-Me H3-K9 and mono-Me H4-K20 throughout the coding region, and a decrease in the levels of methylation of H3-K79. Thus, a proposed model for SIRT1-mediated heterochromatin formation includes deacetylation of histone tails, recruitment and deacetylation of histone H1, and spreading of H3-K79 hypomethylation with resultant silencing.

### Interactions of Sirtuins with methyl-transferase/chromatin-remodeling complexes

In addition to changes in acetylation in the N-terminal domains of histone polypeptides, methylation modification in histone tails also plays an important role in the transition between open and compacted chromatin states. SIRT1 has been shown to interact with a number of methyltransferase/chromatin-remodeling complexes, suggesting a role of SIRT1 at this level of transcriptional regulation.

Methylation at K27 of histone H3 is a repressive “mark,” catalyzed by the SET-domain containing Enhancer of Zeste protein-2, Ezh2, in Polycomb Repressive Complexes (PRCs). Ezh2 is a histone-lysine methyltransferase with activity dependent upon its association with other components of the PRCs. Recently, SIRT1 has been found to form a complex with isoform 2 of the PRC component Eed [63]. The association of SIRT1 with PRCs produces the differential histone substrate specificities of distinct PRCs. Although full functional role of this association is not yet understood, Ezh2 and SIRT1 both are highly expressed in prostate cancer and are associated with prostate cancer progression [64–67] (and our unpublished data). Our recent, unpublished data also shows that SIRT1 plays a role in the silencing of TSG transcription in prostate cancer. Collectively, these findings suggest that SIRT1 may interact with, and cooperate with, EzH2 complexes to silence TSG transcription and promote prostate cancer progression.

Cooperation of deacetylases with other enzymes or other co-repressor proteins to remodel chromatin is suggested by the altered recruitment of methyl-CpG-binding proteins to a methylated CpG island after treatment of tumor cells with HDAC inhibitors. Human cells lacking DNA methyltransferase 1 (Dnmt1), but not those lacking Dnmt3b, demonstrate a loss of DNA methylation and an increase in the acetylation levels of Lys-16 on histone H4 at the rRNA genes. SIRT1, which demonstrates a preference for Lys-16 H4, interacts with Dnmt1, and SIRT1 recruitment to the rRNA genes is abrogated in Dnmt1-knockout cells [68]. The observed DNA methylation and chromatin changes at rRNA genes in Dnmt1-knockout cells are associated with a structurally disorganized nucleolus, which is fragmented into small nuclear masses. These findings suggest a role for SIRT1, in concert with Dnmt1, as an epigenetic caretaker for the maintenance of nucleolar structure [68].

H3-K79 hypomethylation is found in transcriptionally-repressed regions in mammal genomes. SIRT1 over-expression results in the reduction of methylated histone H3-K79 at promoters and within coding regions. H3-K79 methylation restricts the spreading of Sir2p in yeast, and thus, methylation of this residue may constitute a “boundary mark,” separating heterochromatic from euchromatic regions. These and other findings suggest that acetylation/deacetylation and methylation of chromatin can be coordinately regulated [62].

Most recently, SIRT1 has been found to directly interact with and deacetylate the histone methyl-transferase SUV39H1 (suppressor of variegation 3–9 homologue 1), increasing its activity. Loss of SIRT1 strongly affects SUV39H1-dependent generation of H3K9me3 and impairs localization of heterochromatin protein 1 [69].

## Other Mechanisms of Transcriptional Control by Sirtuins

Several reports have shown that SIRT1 can suppress gene transcription by binding directly at gene promoters, but the molecular mechanism underlying this activity are not clear. For example, SIRT1 represses the uncoupling protein (UCP) gene *UCP2* by binding directly to the UCP2 promoter. In beta cell lines in which SIRT1 levels are reduced by SiRNA, UCP2 levels are elevated and insulin secretion is blunted. This up-regulation of UCP2 is associated with a failure of cells to increase ATP levels after glucose stimulation. Knock-down of UCP2 restores insulin secretion in cells with reduced SIRT1, indicating that the defect in glucose-stimulated insulin secretion is due to induction of UCP2 by SIRT1 depletion [70].

Over-expression of SIRT7 increases rRNA transcription, whereas its down-regulation decreases rRNA transcription. Interestingly, SIRT7 expression is enriched in tissues with a high proliferative potential, such as liver, spleen, and testis. Conversely, tissues with a low cellular turnover rate, such as skeletal and heart muscle and brain, express low levels of SIRT7. SIRT7 appears to drive ribosome biogenesis in dividing cells, and has been associated with thyroid and breast cancers (62, 63).

## Conclusion, Perspectives and Unanswered Questions

Since the initial identification of p53 in 2001 as the first transcription factor substrate of SIRT1, multiple diverse transcription factors and co-activator/co-repressor proteins have been identified as targets of SIRT1, enabling direct control of transcription by the Sirtuin family members (Table 1). SIRT1 also modulates chromatin structure, and hence can also affect gene regulation through indirect mechanisms. Yet, there are many important aspects of transcriptional regulation by Sirtuins which require further investigation and elucidation. For example, although it is clear that Sirtuin proteins function in a concerted way with methytransferase and chromatin-remodeling complexes, the molecular details underlying this specific mechanism of transcriptional control are unknown.

In this review, we have discussed the molecular regulatory events targeted by Sirtuins in gene transcriptional regulation. However, Sirtuins is subject to multiple post-translational modifications, the molecular roles of which remain largely uncharacterized. Furthermore, as these post-translational modifications are effected by various signaling pathways, this presents yet another level of control of Sirtuins on transcription that remains to be elucidated in detail. As examples, phosphorylation of HuR by Chk2 regulates SIRT1 expression. Depending on its phosphorylation state, HuR can cause SIRT1 mRNA to either be stabilized or degraded [71, 72]. Over-expression of the protein phosphatase CDC14B results in dephosphorylation of SIRT2, with a subsequent decrease in the levels of SIRT2 protein [73]. Inhibition of PI_3_Kinase activity alters SIRT1 subcellular localization [25] and SIRT1 protein levels (our unpublished data). Precisely how SIRT1 is regulated by PI_3_Kinase or by other post-translational modification, with respect to compartmental localization or protein levels, and its ultimate effects on transcriptional activity, are unknown. Whether post-translational modifications of Sirtuins affect the acetylation status of promoter elements in a gene-specific manner also remains to be determined. Certainly, identification of physiologically-relevant signaling pathways which induce the activation or the inactivation of Sirtuins, and their consequences on transcription, will be important to our understanding of this central transcriptional regulator.

Another perhaps surprising but major question which remains unanswered is whether SIRT1 is a tumor suppressor or, instead, an oncogene. While multiple lines of evidence suggest that Sirtuins protect against cancer development [16, 17, 74], several forms of cancer are characterized by elevated levels of SIRT1 in the tumor cells, and these malignant cells may be dependent upon SIRT1 for their proliferation and survival (Sirtuin “addiction”) [61, 67, 75–77]. For example, in prostate cancer, SIRT1 is required for androgen antagonist-mediated transcriptional repression and growth suppression [17], and deacetylation of the AR by SIRT1 inactivates its ability to transform prostate cells [16]. Conversely, SIRT1 has been shown to be over-expressed in cancer cells and to promote cancer cell survival [75, 77]. Our unpublished data demonstrates that SIRT1 silences specific tumor suppressor genes in refractory prostate cancer cells, that SIRT1 is over-expressed both in the nucleus and cytoplasm of prostate cancer cells, and that this over-expression is associated with prostate cancer progression. These seemingly conflicting data suggest that SIRT1 may play dual roles in cancer progression, depending upon the physiological conditions of the organism, or the stage of the cancer. For example, in the case of prostate cancer, we hypothesize that during the adult development of prostate, as the expression level of SIRT1 decreases with aging, this promotes replicative senescence and protects against tumorigenesis (although paradoxically also increasing the sensitivity of this tissue to androgen stimulation). At this point, SIRT1 serves as a tumor suppressor. However, with the development of cancer, loss of tumor-suppressor genes such as HIC1 and p53, which regulate SIRT1 expression, may then lead to the over-expression of SIRT1. Through consequent SIRT1-induced silencing of tumor suppressor genes, SIRT1 then plays a role in cancer progression, promoting oncogenesis. Future work in this area, utilizing tissue—restricted transgenic or gene knock-out mouse models is need to fully understand such dichotomous roles for SIRT1 in cancer protection and progression.

Poised as they are, at the critical intersection of processes as diverse as aging, cancer susceptibility and metabolic regulation, a clearer mechanistic understanding of the central role that Sirtuins play at multiple levels in transcriptional control is of great importance.

## Figures and Tables

**Table 1:** Transcription factors and their coregulators are listed that have been shown to be deactylated by Sirtuins.

Substrate	Sirtuins	Functions and references
FOXO1	SIRT1	Inhibit apoptosis (6); Increase glucose release (7)
	SIRT2	regulator of adipocyte differentiation (22)
FOXO3	SIRT1	Induce cell cycle arrest and inhibit apoptosis (8); Inhibit Apoptosis (9)
	SIRT2	Promote cell death, induce cell cycle arrest (41)
FOXO4	SIRT1	Promote cellular survival and increase life span (10)
p53	SIRT1	Inhibit apoptosis (11, 12); antagonize PML/p53-induced cellular senescence (13)
p73	SIRT1	Inhibit apoptosis (38)
NFκB	SIRT1	Increase TNFalpha induced apoptosis (14)
PPARγ	SIRT1	Promotes fat mobilization (15)
AR	SIRT1	Suppression of AR mediated transcription (16, 17)
Smad7	SIRT1	Attenuates Smad7-induced mesangial cell apoptosis (32)
E2F	SIRT1	Inhibition of apoptosis (39)
Rb	SIRT1	Suppressor of Rb (40)
P300	SIRT1	Suppressor of p300 (44)
PGC-1	SIRT1	Activates gluconeogenesis and represses glycolysis (45,46), protected diet-induced-obesity and insulin resistance [47]; promote the activation of mitochondrial fatty acid oxidation genes [48] and regulate cellular oxygen consumption [49]
	SIRT3	Reactive oxygen species production and increase cellular respiration (50)
CTP1/CTP2	SIRT1	Stimulates CTIP-dependent transcriptional repression (51, 52)
bHLH and HES	SIRT1	Enhance the transcription suppression in metazoan development (53)
HIC1	SIRT1	Positively control HIC1 transcriptional repression activity (54)
Tat	SIRT1	Transcriptional coactivator during Tat transactivation (56)
